# Constitutional DNA Polymorphisms Associated with the Plasma Imatinib Concentration in Chronic Myeloid Leukemia Patients

**DOI:** 10.3390/pharmaceutics16060834

**Published:** 2024-06-19

**Authors:** Heriberto Bruzzoni-Giovanelli, Habib Zouali, Mourad Sahbatou, Benjamin Maneglier, Jean-Michel Cayuela, Angelita Rebollo, Gustavo H. Marin, Daniela Geromin, Carole Tomczak, Antonio Alberdi, Jean-Francois Deleuze, Philippe Rousselot

**Affiliations:** 1 Centre d’Investigation Clinique, 1427 Inserm/AP-HP, Hôpital Saint-Louis, Université Paris Cité, 75010 Paris, France; 2 Fondation Jean Dausset-Centre d’Étude du Polymorphisme Humain (CEPH), 27 Rue Juliette Dodu, 75010 Paris, France; 3 Département de Pharmacologie, Centre Hospitalier de Versailles, 78150 Le Chesnay, France; 4 Département d’Hématologie et Biologie Moléculaire et EA3518, Hôpital Saint-Louis, AP-HP, 75010 Paris, France; 5 UTCBS, INSERM U1267-CNRS UMR8258, Faculté de Pharmacie, Université de Paris, 4 Avenue de l’Observatoire, CEDEX 06, 75270 Paris, France; 6 CUFAR, Farmacologia Básica, CONICET—FCMLP, Universidad Nacional de La Plata, 60 & 120, La Plata 1900, Argentina; 7 UMS Saint-Louis US53/UAR2030, Institut de Recherche Saint Louis, Plateforme Technologique Centre Hayem, Hôpital Saint-Louis, Université Paris Cite—INSERM—CNRS, 1 Av Claude Vellefaux, CEDEX 10, 75475 Paris, France; antonio.alberdi@u-paris.fr; 8 Centre National de Recherche en Génomique Humaine, Institut François Jacob, CEA, Université Paris Saclay, CNRGH, 91190 Evry, France; 9 Département d’Hématologie, Centre Hospitalier de Versailles, 78157 Le Chesnay, France; 10 UMR1184, Département IDMIT, Commissariat à L’énergie Atomique et aux Energies Alternatives, Université de Versailles Saint-Quentin-en-Yvelines Paris-Saclay, 92265 Fontenay-Aux-Roses, France

**Keywords:** imatinib, plasma concentration, SNP, exome, pharmacogenetics, polymorphism, chronic myeloid leukemia, CML

## Abstract

The tyrosine kinase Inhibitor (TKI) imatinib is approved for the treatment of the chronic phase of chronic myeloid leukemia (CP-CML). Pharmacokinetic studies have highlighted the importance of inter-patient variability on imatinib plasma trough concentrations (ima[C]min). In the OPTIM-imatinib trial, we demonstrated that therapeutic drug monitoring (TDM) is able to improve the molecular response of CP-CML patients treated with imatinib. Here, we analyzed the constitutional exomes and RNAseq data of these patients. We performed an association analysis between the constitutional genetic variants of the patients and their ima[C]min, measured after 12 weeks of treatment with 400 mg once daily. Using linear regression, we identified 50 SNPs that showed excess heterozygosity depending on the ima[C]min. Ten SNPs were from non-coding sequences, and among the 40 remaining, 30 (from 25 genes) could be split into two categories. The first group of 16 SNPs concerns genes encoding extracellular matrix, cell junction, and membrane proteins. Coincidentally, cell adhesion proteins were also identified by RNA-seq as being overexpressed in patients with high ima[C]min. The other group of 14 SNPs were from genes encoding proteins involved in transcription/translation. Although most of the SNPs are intronic variants (28), we also identified missense (3), synonymous (4), 5′/3′ (2), splicing (1), and upstream (4) variants. A haplotype analysis of four genes showed a significant association with high ima[C]min. None of the SNPs were significantly associated with the response. In conclusion, we identified a number of ima[C]min-associated SNPs, most of which correspond to genes encoding proteins that could play a role in the diffusion and transit of imatinib through membranes or epithelial barriers.

## 1. Introduction

Chronic myeloid leukemia (CML) is a myeloproliferative disorder associated with a translocation that results in a BCR::ABL1 fusion gene with enhanced and deregulated tyrosine kinase activity [1]. Imatinib was the first tyrosine kinase inhibitor (TKI) approved for CP-CML at a dose of 400 mg once daily [2]. Imatinib dose optimization has been evaluated in several prospective clinical studies that tested the use of high doses (from 600 mg to 800 mg daily). The Tyrosine Kinase Inhibitor Optimization and Selectivity (TOPS) study randomly compared 400 mg/day of imatinib (*n* = 157) to 800 mg/day of imatinib (*n* = 319) in patients newly diagnosed with Philadelphia chromosome-positive CML in the chronic phase (CP-CML). After a median follow-up of 42 months, major molecular response (MMR) rates were similar for the two arms, without differences in event-free survival (EFS), progression-free survival (PFS), or overall survival (OS). Of note, patients who were able to tolerate ≥ 600 mg/day of imatinib in the first year of treatment showed a faster response and higher response rates [3]. The phase II randomized study of the SWOG (SWOG S0325) also compared 400 mg/day to 800 mg/day of imatinib in 153 adult patients with CP-CML. The molecular response (MR) at 12 months was greater in the IM800 arm, but toxicity was higher and no long-term follow-up has been reported [4]. In both the French SPIRIT [5] and German CMLIV [6] randomized phase III studies, the 600-mg arm demonstrated no superiority in terms of molecular response, despite the more rapid achievement of a strong molecular response in the CMLIV trial. A systematic review and meta-analysis of randomized controlled trials comparing frontline treatment with 400 mg of imatinib daily versus high doses concluded that these strategies increase toxicity while providing only minimal therapeutic advantage [7,8]. Pharmacokinetic studies have highlighted the importance of inter-patient variability in imatinib plasma trough concentrations (ima[C]min), which varied from 55 to 106% among patients for a given dose [9]. The ima[C]min obtained after dose adjustment correlated with pharmacodynamic responses and it has been suggested that a threshold of 1000 ng/mL is associated with an improved molecular response in patients treated with imatinib [9,10,11]. In the prospective randomized OPTIM-imatinib trial, the TDM value was assessed in patients with CP-CML treated with 400 mg of imatinib daily as first-line therapy [12]. The TDM strategy resulted in a significant increase in ima[C]min values and, after 12 months, significantly improved the cumulative major molecular response (MMR) rate of patients with a low initial ima[C]min.

Although we and others have focused on DNA polymorphisms associated with the response to imatinib [13,14,15], very few genetic studies have been performed to explore associations between SNPs and imatinib plasma concentrations [16,17,18,19], with none in relation to RNA expression. Moreover, the SNP studies were limited to the candidate-gene approach, which has several limitations, such as a reliance on a priori hypotheses and frequent arbitrary choices, which have been largely detailed [20,21]. We performed exome sequencing of constitutional DNA and RNA-seq of patients included in the OPTIM-imatinib trial to identify genetic variants associated with ima[C]min in coding regions without a priori selection. The ima[C]min was measured after 12 weeks of treatment at 400 mg daily and before any dose adjustment, as planned in the protocol. These values were then analyzed as a function of the genetic data obtained from each patient.

## 2. Materials and Methods

For detailed information please refer to Supplementary Methods and Data.

### 2.1. Informed Consent 

Informed consent for the genetic and pharmacokinetics analyses was previously obtained from the patients participating in the OPTIM-imatinib clinical trial (EudraCT number 2008-006854-17, NCT02896842) [12].

### 2.2. Measurement of Residual Plasma Imatinib Concentrations (ima[C]min)

The initial ima[C]min of each patient was evaluated after 12 weeks of treatment at a dose of 400 mg daily, at the time of arm assignation in the OPTIM-imatinib study (before any dose adaptation). The ima[C]min was centrally determined using chromatography-tandem mass spectrometry, as previously described [12].

### 2.3. Whole-Exome Sequencing (WES)

Constitutional DNA from peripheral blood mononuclear cell (PBMC) samples was extracted from 114 patients participating in the OPTIM-imatinib trial. In total, 100 samples passed all quality controls and were sequenced on the Illumina sequencing platform at the Centre National de Recherche en Génomique Humaine (CNRGH).

### 2.4. RNA-Sequencing

RNA from 61 patients was extracted and sequenced on the Illumina sequencing platform at the CNRGH.

### 2.5. Association Studies

The principal component analysis (PCA) using PLINK software (v1.90b3f 64-bit, 2 Mar 2015) [22] revealed that 92 of the 100 samples (65 men and 27 women) passed the filters and quality control (QC) checks, and they were subsequently included in the association analysis (Supplementary Figure S1).

### 2.6. Linear Regression Analysis

The quantitative trait ima[C]min was tested for its association with genetic variants using Plink software, considering either asymptotic (using the likelihood ratio test and Wald test) or empirical significance values. Standard linear regression was performed by estimating the additive genetic model (the additive effects of SNPs), i.e., the dose-dependent effect of the minor alleles.

## 3. Results

### 3.1. Patient Population

The median age of CML patients was 65 years (63 years for men and 68 years for women). The ima[C]min of patients whose DNA was sequenced ranged from 236 to 2292 ng/mL, with a mean of 936.9 ng/mL and a median of 802.5 ng/mL.

After principal component analysis and the removal of replicates, 92 patients were retained for the analysis (65 men and 27 women, Figure S1).

### 3.2. Association Analysis in Binary Mode

Based on a previous study suggesting that a concentration of 1000 ng/mL may be associated with the response [10], two groups of CML patients were considered based on their initial ima[C]min. The first group included 35 patients with plasma imatinib concentrations > 1000 ng/mL and the second group included 57 patients with plasma imatinib concentrations < 1000 ng/mL. Fisher’s exact test was performed to test for associations between these groups.
**Phenotype Code****Phenotype****Samples (*n*)**Binary modeConcentration > 1000 ng/mL (*n =* 35)92Concentration < 1000 ng/mL (*n =* 57)

The association analysis comparing these two groups identified 281 SNPs (including 156 from known genes) associated with an ima[C]min > 1000 ng/mL (supplementary Tables S1.1 and S1.2). The *p*-values were not corrected for multiple testing and values ≤ 0.001 were considered suggestive. The SNPs were identified using a mean depth cut-off of four, i.e., each SNP should be covered by at least four reads.

As we did not find an association between ima[C]min and the MMR rate in the OPTIM-imatinib clinical trial, we conducted the association study between genotypes and ima[C]min using linear regression analysis.

### 3.3. Linear Regression Analysis of ima[C]min

We performed standard linear regressions using PLINK software. Plasma concentration was considered to be a quantitative trait. The additive effects of SNPs (ADD model) were estimated.

The linear regression analysis used to test the association between SNPs and ima[C]min identified 845 SNPs, of which 479 were from known genes with *p*-values ≤ 10^−3^ (Table 1). As for the binary analysis, the *p*-values calculated from the applied standard regression were not corrected for multiple testing. The same criteria as those applied to the binary association study were used, i.e., uncorrected *p*-values ≤ 0.001 and a mean depth cut-off of four reads.

Details of the 845 SNPs associated with ima[C]min (with a *p*-value < 10^−3^ and a mean depth of sequencing > 4) are presented in Tables S2.1 and S2.2. The effect size of a single SNP upon ima[C]min was expressed by estimating the beta coefficient, which represents the strength of the relationship between the two [23]. The values of the beta coefficient for each SNP are showed in Tables S2.1 and S2.2 (in the ninth column). The direction of the relationship could be positive or negative, i.e., SNPs associated with a high or low ima[C]min (Supplementary Methods and Data, “Linear regression analysis”). The beta coefficient values varied from 189.9 to 1406 for 782 of the 845 SNPs (92.54%, positive direction) and from −450 to −200.2 for the remaining 63 SNPs (7.46%, negative direction). A Manhattan plot of the selected genes associated with the ima[C]min is shown in Figure S2. In total, 62 SNPs corresponding to 47 genes were common to both the linear regression and binary analyses (Tables S3.1 and S3.2).

Examination of the genotypes identified for the 92 patients and the corresponding ima[C]min showed that certain SNPs exhibited an excess heterozygosity rate depending on the imatinib concentration. We next focused on the 130 SNPs identified by linear regression (Table S4) with *p*-value ≤ 10^−4^ and a mean sequencing depth > 4, and analyzed their heterozygosity in our population (as an indicator of the number of patients affected by each polymorphism). We confirmed excess heterozygosity rates for a large number of SNPs based on individual ima[C]min values (Table S5). Moreover, we decided to consider polymorphisms that concerned at least 7% of the patients (also excluding the SNPs of sex chromosomes). Under these conditions, we identified 50 SNPs, 10 from non-coding sequences and 40 from 34 genes, that were associated with ima[C]min (Table 2). Among these 40 SNPs, 16 were found in 11 genes encoding extracellular and membrane proteins (Table 2, highlighted in yellow). Among these genes, we found Muc2, encoding a glycoprotein produced and secreted by epithelial cells that forms an insoluble mucous barrier protecting the gut lumen; PLEKHA7, encoding a protein involved in epithelial cell–cell adhesion; EXOC6B, encoding a component of exocyst, a multimeric protein complex necessary for exocytosis; LEPROTL1, whose protein is involved in late endosome-to-vacuole transport via the multivesicular body sorting pathway; SSC5D, predicted to be associated with fibronectin- and collagen-containing extracellular matrix regulation and interleukin-8 production; IL12RB2, encoding a transmembrane protein identified as a subunit of the interleukin 12 receptor complex; and the TCTN2 gene, encoding a membrane protein for which mutations are associated with ciliopathies. We also found EGFLAM, encoding a protein predicted to enable Ca^++^ glycosaminoglycan binding activity, involved in the organization of the extracellular matrix and positive regulation of cell–substrate adhesion. The identified variant of EGFLAM results in a missense mutation. In addition, we identified 14 SNPs (35%) from 14 genes coding for proteins associated with transcription and translation (highlighted in green). Among the rest, five SNPs correspond to five kinases, two to one phosphatase, one to a centrosome protein, one to a cytochrome, and one to a catabolic enzyme of GABA.

Among the 50 SNPs identified in Table 2, three correspond to missense variants, four are synonymous variants, two are 3′ or 5′ prime UTR variants, one is a splice region variant, three are upstream gene variants, twenty-seven are intronic variants, eight are found in intergenic regions, and two are non-coding transcript exon variants.

**Figure 1:**
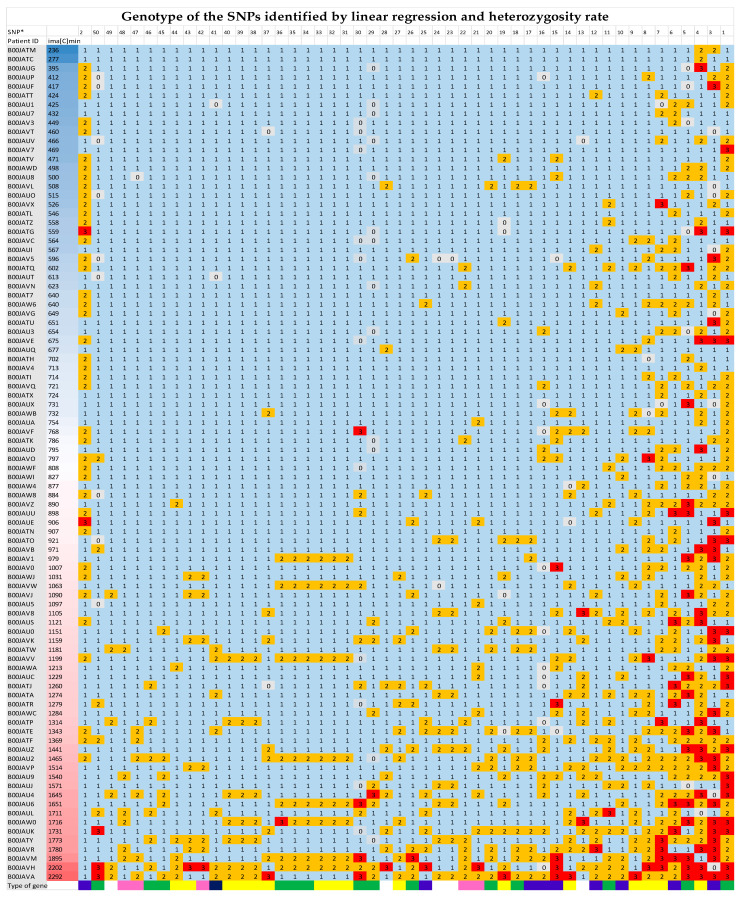
HeatMap of the 50 SNPs (from Table 2) showing an excess heterozygosity rate sorted by ima[C]min. The patients (1st column) are classified according to ima[C]min (2nd column). The 3rd to 53rd columns represent the genotype of the 50 SNPs, classified according to heterozygosity rate from the lowest on the left to the highest on the right (in the first arrow SNPs are numerated from 1 to 50 according to the Table 2: SNP* column). Cell color code: red (code 3) represents patients homozygous for the minor allele, orange (code 2) represents heterozygous patients, sky blue (code 1) major allele, and grey (code 0) data non-available. As shown in the figure, the number of patients heterozygous or homozygous for the minor allele for each SNP gradually increases with the ima[C]min. An exception is observed for the SNP rs614461 (SNP* 2, 3rd column), for which the excess heterozygosity rate is associated with a low ima[C]min. The last row shows the type of gene corresponding to each SNP (color coded according to Table 2).

The excess of heterozygosity rate for the 50 SNPs presented in Table 2 in function of ima[C]min can be clearly visualized in the Figure 1.

### 3.4. Haplotype Analysis

Certain SNPs identified using linear regression show a significantly suggestive association and belong to the same gene. As the haplotype is more informative than a single marker, we conducted a haplotypic association test using Haploview software V4.2 [16]. Four genes (NIPBL, TCTN2, BPGM, and EXOC6B) with at least two SNPs each were tested for haplotype association. The SNP rs969477092, located on the NIPBL gene, was used to generate a linkage disequilibrium (LD) plot characterizing haplotype blocks over a distance of 50 kb (Figure S3.1). A pairwise analysis of the linkage disequilibrium (LD) between the NIPBL SNPs is presented in the Supplementary Materials and showed seven LD haplotype blocks. Block 5 contained six SNPs, five of which (rs969477092, rs775871417, rs1170486575, chr5:36975684, and chr5:36975687) are associated with ima[C]min. These SNPs are in LD, as determined by r2 value obtained with Haploview. Both D’ and r2 statistics provided evidence with statistical confidence (LOD > 2) for strong LD (D’ = 1, r2 = 0.59–1).

Moreover, Haploview software generates haplotypes and their population frequencies [24]. As affection status is included in the input file, Haploview also calculates simple χ^2^ statistics (for ima[C]min less than or greater than 1000 ng/mL) for each haplotype in each block, which can be used for association studies. The results of the haplotype association are summarized in Table 3. The haplotype association analysis identified the haplotype “CAGAAC” of LD Block 5 (*p*-value = 6 × 10^−4^) for patients with an ima[C]min > 1000 ng/mL.

Haplotypes of three other genes from Table 2 were found to be associated with an ima[C]min > 1000 ng/mL: “CGAAC” in *BPGM* (rs1173882568 and rs1396579274, *p*-value = 6 × 10^−4^); “AAA” in *TCTN2* (rs3748271, rs11057329, and rs10846543, *p*-value = 3.2 × 10^−3^), and “GCC” in *EXOC6B* (rs61736520 and rs11689707, *p*-value = 3.1 × 10^−03^) (Figure S3).

### 3.5. Transcriptomic Changes in Patients with Plasma ima[C]min > 1000 ng/mL

Differential expression (DE) analyses were conducted using the *DESeq2* tool. A comparison of ima[C]min > 1000 ng/mL (20 patients) versus ima[C]min < 1000 ng/mL (41 patients) showed there to be no differentially expressed genes (DEGs) that met our criteria (BH-adjusted *p*-values ≤ 0.05 and |log2 FC (fold-change)| > 1). We plotted a histogram of the *p*-values, showing that they had not been correctly computed (Figure S4.1). The new BH-adjusted *p*-values were re-estimated using fdrtool and a new histogram was plotted (Figure S4.2). The results for the DEGs were visualized using a volcano plot (Figure S5). The DEGs were considered to be up- or downregulated for fold changes > 1 or <1, respectively. In total, we identified 439 DEGs (up/down = 257/182; Figure S6) after correction of the *p*-values (Table S6).

Among the DEGs, six genes (*MPV17L*, *LSAMP*, *EPB41L4B*, *CLDN34*, *EMP2*, and *FREM3*) code for membrane proteins, of which the last four play a role in bicellular tight junction assembly and cell adhesion. The proteins encoded by the first four genes were found to be upregulated. Two DEGs coding for two extracellular matrix proteins (metalloproteinases *ADAMTS2* and A*DAMTS3*) with thrombospondin Type 1 Motif 2–3 were also shown to be upregulated. The SNP (rs1110514) located in the *ADAMTS2* gene was also shown to be associated with ima[C]min in the exome analysis. Moreover, eight DEGs coding for immunoglobulins were also identified. Three of them were found to be upregulated. In addition, three other upregulated genes were found: the *MPL* gene encoding the thrombopoietin receptor associated with myeloproliferative disorder, the *LIF* gene, which is an inducer of hematopoietic differentiation in normal and myeloid leukemia cells, and the *CAMK2N2* gene, an inhibitor of protein kinase II.

To gain a deeper understanding of the differences in the cellular transcriptome, we conducted a gene set enrichment analysis (GSEA, see Supplementary Methods). The GSEA of the normalized data showed the top 50 ranked genes that overlap between CML patients with an ima[C]min > 1000 and those with an ima[C]min < 1000 ng/mL. The GSEA showed the distinct on/off switching of genes, suggesting a pattern of upregulated/downregulated genes associated with an ima[C]min > 1000 ng/mL (Figure S6).

## 4. Discussion

In this study, we identified 130 SNPs, 103 of which correspond to coding genes, associated with residual plasma concentrations of imatinib (*p* ≤ 10^−4^) in CML-CP patients measured after 12 weeks of treatment at 400 mg per day. By analyzing the heterozygosity rate of the identified SNPs, we found 50 SNPs that involved a minimum of 7% of patients. Ten are from non-coding sequences. Examination of the function and localization of proteins encoded by the remaining 40 SNPs (34 genes) made it possible to classify 30 of them into two main categories. The first category includes 16 SNPs from 11 genes coding for extracellular and membrane proteins involved in epithelial cell adhesion, the formation of the mucous barrier in the gut lumen, exocytosis, and endosomal vacuole transport (Muc2, PLEKHA7, EXOC6B, and LEPROTL1, Table 2, highlighted in yellow). Two of these SNPs from two genes (SSC5D and IL12RB2) are also involved in interleukin signaling. Another encoded protein from this group of genes is EGFLAM, which is involved in the regulation of cell–substrate adhesion and extracellular matrix organization. It is worth highlighting that the identified variant (rs35767836) results in a missense mutation. The second main group includes 14 SNPs from 14 genes encoding proteins associated with transcription and translation (Table 2, highlighted in green). Among the remaining SNPs, we found five corresponding to protein kinases, three that are components of mitotic spindle organization, and one (rs35840993) corresponding to cytochrome CYP4F3, a gene previously shown to be associated with the cytogenetic response to imatinib [25].

We conducted a haplotype analysis for four genes, for which at least two SNPs were identified. This analysis revealed a block of five SNPs located in the NIPBL gene that are in LD with each other and associated with a high ima[C]min. NIPBL is a sister chromatid cohesion protein involved in the regulation of development.

As already mentioned, very few studies have previously been performed to identify genetic variants potentially associated with ima[C]min. These studies were conducted on a small number of patients using the candidate-gene approach [16,17,18,19]. However, the candidate-gene approach presents several limitations: the choice of candidate genes may be inappropriate, the causative genes may be either upstream or downstream in signaling pathways, the selected SNPs may provide incomplete coverage of the gene, most studies were underpowered, and importantly, these studies relied on prior hypotheses, which precludes the discovery of genetic variants in previously unknown pathways. On the contrary, we chose to perform whole-exome sequencing of constitutional DNA and RNA-seq analysis of patients without a priori selection to identify any genetic variant associated with ima[C]min.

In our clinical trial, no association was found between the plasma ima[C]min and MMR rate at 12 months for unadjusted-dose patients. Consistent with this finding, we found no common SNPs in our association analysis between plasma ima[C]min and the MMR rate. We also did not find any SNPs that we and others had previously found to be associated with the molecular response [13], with the exception of CYP4F3, which was reported in 2004 but has never been retested [25]. Furthermore, recently published studies focusing on genes selected for their previously claimed pharmacological functions (such as CYP3A4, CYP3A5, ABCB1, ABCG2, and SCL22A1) have failed to demonstrate an association with imatinib clearance [16], whereas the hemoglobin concentration and estimated glomerular filtration rate did [18]. Thus, the question has been raised as to whether measuring plasma imatinib trough levels is an appropriate means to predict the response of CML patients [26].

From our data, it can be hypothesized that certain patients may have a genetic background that is less favorable for imatinib clearance outside of the genes known to be directly involved in imatinib pharmacology. A low initial plasma imatinib level may be a poor response predictor, rectifiable with dose adaptation, as shown in our OPTIM-imatinib trial (in which a TDM strategy of increasing daily oral imatinib doses improved the MMR rate at 12 months from 39 to 67% for treated CML patients) [12]. On the other hand, initially high ima[C]min may not be a good predictor of a good MMR rate, but as expected, a high initial ima[C]min may be associated with a greater frequency of adverse events (Table S7). The correlation between plasma imatinib concentration and patient response needs to be studied in depth. It is important to emphasize that the plasma levels of a drug do not always reflect its intracellular concentration. Elevated plasma but not intracellular concentrations have been reported after liposomal daunorubicin infusion versus conventional daunorubicin treatment in adults with acute myeloid leukemia [27]. It has also been shown that there is no significant correlation between the anti-HIV drug Darunavir plasma trough levels and its concentration in isolated PBMCs [28]. Therefore, and taking into account the presence of transporters and cytochromes in the cell membrane of white blood cells, it may be more relevant to study whether there is a correlation between the concentration of imatinib measured in isolated PBMCs and the molecular response of CML-CP patients.

Although most of the SNPs identified in the present study are intronic variants, we cannot exclude that they may have an effect on gene expression, some being intronic sequences within regulatory regions (splicing regions).

In addition, the correlation between clinical phenotype and identified SNPs in association studies does not necessarily constitute a causal link. Moreover, as in most association studies of complex traits, the effect size of each SNP is relatively small (as shown by the beta values in Table S2), but the effect of each single associated SNP contributes to the overall association [21].

At the same time, it is intriguing that most of the SNPs correspond to genes whose products could affect the cell membrane or the passage of imatinib through the epithelial barrier. Interestingly, the RNAseq analysis identified a number of extracellular and membrane proteins (some of which play a role in cell adhesion) as being upregulated. On the other hand, given the current state of knowledge, the potential biological significance of the other group of genes (associated with transcription/translation and mitosis) is much less clear. Of note, the expression of the only cytochrome identified in our study of SNP association (CYP4F3) was not found to be downregulated.

## 5. Conclusions

This is the largest study attempting to identify genetic variants associated with plasma imatinib levels in CP-CML patients and the only one without an a priori hypothesis. We identified a number of SNPs in genes not previously described to be associated with imatinib clearance. These SNPs belong to genes potentially involved in the passage of imatinib through biological barriers and, consequently, its concentration in various compartments. Our data support the hypothesis that genetic background, apart from the classically invoked gene, hemoglobin concentration, and glomerular filtration rate, may play a role in imatinib exposure levels in CP-CML patients.

## Figures and Tables

**Table 1:** SNPs identified by linear regression to be associated with the ima[C]min phenotype.

*p*-Value ≤ 10^−3^		*p*-Value ≤ 10^−4^		*p*-Value ≤ 10^−5^		*p*-Value ≤ 10^−6^	
SNPs (*n*)	Genes (*n*)	SNPs (*n*)	Genes (*n*)	SNPs (*n*)	Genes (*n*)	SNPs (*n*)	Genes (*n*)
845	479	130	76	24	11	4	1

**Table 2:** 50 SNPs identified by linear regression and ordered by heterozygosity rate. List of 50 SNPs associated with ima[C]min identified using linear regression (SNPs with *p*-value ≤ 10^−4^, a mean sequencing depth > 4, and concerning at least 7% of patients). The SNPs are sorted according to heterozygosity rate (highest to lowest) and numbered from 1 to 50 (column SNP*). The cells are highlighted as follows: yellow, 16 SNPs from 11 genes encoding extracellular and membrane proteins; green, 14 SNPs from 14 genes encoding proteins associated with transcription and translation; violet, 5 SNPs from five genes encoding kinases; white, various SNPs; and dark blue, 10 SNPs from non-coding sequences.

SNP	SNP*	Chromosome	Gene	Minor Allele A1	Frequent Allele A2	Homozygote A1 (*n*)	Heterozygotes (*n*)	Homozygote A2 (*n*)	*p*-Value Linear Regression	Variant Type
rs773894	1	19	**NWD1**	G	C	17	50	25	3.60 × 10^−5^	intron_variant
rs614461	2	1	**ESPNP**	G	C	2	41	49	6.42 × 10^−5^	non_coding_transcript_exon_variant
rs41500945	3	11	**MUC2**	T	C	14	36	41	1.35 × 10^−5^	intron_variant
rs7781826	4	7	**NC_000007.14:144435107:C:A**	C	A	7	35	50	2.49 × 10^−5^	intergenic_region
rs56156123	5	11	**PLEKHA7**	T	C	6	29	55	6.16 × 10^−5^	intron_variant
rs34933848	6	19	**SSC5D**	A	G	3	27	60	5.66 × 10^−5^	intron_variant
rs7895028	7	10	**NC_000010.11:100656250:T:C**	C	T	22	20	41	1.55 × 10^−5^	intergenic_region
rs10863292	8	1	**ESRRG**	C	T	15	19	52	1.40 × 10^−5^	intron_variant
rs61736520	9	2	**EXOC6B**	C	T	1	19	72	8.30 × 10^−5^	synonymous_variant
rs796983937	10	6	**TCP10L2**	G	C	0	17	75	2.46 × 10^−5^	non_coding_transcript_exon_variant
rs112924481	11	1	**ZZZ3**	C	T	1	16	75	1.39 × 10^−5^	intron_variant
rs528965019	12	14	**NC_000014.9:60397689:A:G,NC_000014.9:60397689:A:T**	T	A	1	15	76	4.44 × 10^−5^	intergenic_region
rs151604	13	6	**CEP43**	T	C	2	14	75	7.65 × 10^−6^	intergenic_region
rs11689707	14	2	**EXOC6B**	C	A	1	14	75	3.01 × 10^−5^	intron_variant
rs368063	15	8	**LOC100507403 **	C	G	5	12	73	7.39 × 10^−5^	intergenic_region
rs1490556064	16	5	**HCN1-EMB**	A	C	0	12	70	6.05 × 10^−5^	intergenic_region
rs7170580	17	15	**NC_000015.10:44784456:G:C,NC_000015.10:44784456:G:T**	C	G	0	12	80	8.92 × 10^−5^	intergenic_region
rs17588988	18	15	**TRIM69**	G	A	0	11	81	5.18 × 10^−5^	missense_variant
rs16876504	19	8	**LEPROTL1**	G	A	1	10	77	8.38 × 10^−5^	intron_variant
rs17229438	20	15	**MIR10393**	A	G	0	10	82	2.13 × 10^−5^	upstream_gene_variant
rs55806342	21	10	**PRKG1**	A	G	1	10	81	6.79 × 10^−6^	synonymous_variant
rs116049705	22	1	**AK5**	A	G	0	10	82	3.78 × 10^−5^	5_prime_UTR_variant
rs1173882568	23	7	**BPGM**	T	C	0	10	81	7.76 × 10^−5^	intron_variant
rs1396579274	24	7	**BPGM**	T	G	0	10	80	7.97 × 10^−5^	intron_variant
rs62059984	25	17	**NC_000017.11:14641601:G:A**	A	G	1	9	82	1.43 × 10^−5^	intergenic_region
rs79981660	26	11	**YAP1**	A	G	1	9	82	2.66 × 10^−5^	intron_variant
rs142214363	27	3	**IGSF10**	G	A	0	9	83	5.28 × 10^−5^	synonymous_variant
rs79390594	28	16	**ABAT**	G	A	1	8	83	9.65 × 10^−5^	intron_variant
rs3748271	29	12	**TCTN2**	G	A	0	8	84	7.10 × 10^−5^	intron_variant
rs11057329	30	12	**TCTN2**	T	A	0	8	84	7.10 × 10^−5^	intron_variant
rs10846543	31	12	**TCTN2**	G	A	0	8	84	7.10 × 10^−5^	intron_variant
rs10773019	32	12	**DDX55**	G	A	0	8	84	7.10 × 10^−5^	missense_variant
rs3704	33	12	**EIF2B1**	T	G	0	8	84	7.10 × 10^−5^	intron_variant
rs978263	34	17	**CBX1**	A	G	4	7	70	2.02 × 10^−5^	upstream_gene_variant
rs12694421	35	2	**DIRC3**	T	A	1	7	71	1.55 × 10^−5^	intron_variant
rs10773020	36	12	**GTF2H3**	G	T	1	7	84	8.34 × 10^−5^	upstream_gene_variant
rs6662789	37	1	**IL12RB2**	A	G	1	7	82	4.13 × 10^−5^	intron_variant
rs969477092	38	5	**NIPBL**	T	C	0	7	85	2.04 × 10^−7^	intron_variant
rs775871417	39	5	**NIPBL**	T	A	0	7	85	2.04 × 10^−7^	intron_variant
rs1170486575	40	5	**NIPBL**	T	G	0	7	85	2.04 × 10^−7^	intron_variant
rs1346918408	41	16	**NC_000016.10:33337669:T:C**	C	T	0	7	83	3.24 × 10^−5^	intergenic_region
rs16845711	42	2	**STK17B**	G	A	1	6	85	7.62 × 10^−5^	splice_region_variant&intron_variant
rs16841010	43	2	**DNAH7**	T	C	1	6	85	7.62 × 10^−5^	intron_variant
rs35767836	44	5	**EGFLAM**	C	G	0	6	86	4.99 × 10^−6^	missense_variant
rs41269273	45	6	**H4C12**	T	A	0	6	86	6.32 × 10^−5^	synonymous_variant
rs78352502	46	14	**RPL10L**	T	C	0	6	86	6.88 × 10^−5^	upstream_gene_variant
chr9:27454985	47	9	**MOB3B**	T	A	0	6	85	2.08 × 10^−5^	intron_variant
rs45611038	48	10	**PRKCQ**	A	G	0	6	86	4.41 × 10^−5^	intron_variant
rs35840993	49	19	**CYP4F3**	G	A	0	6	86	6.91 × 10^−5^	3_prime_UTR_variant
rs2178045	50	7	**AUTS2**	A	G	3	5	75	3.37 × 10^−6^	intron_variant

**Table 3:** Haplotypic association from SNPs in the NIPBL gene with ima[C]min.

Block	Haplotype	Freq.	ima[C]min > 1000 ng/mL Ratio Counts	ima[C]min < 1000 ng/mL Ratio Counts	ima[C]min > 1000 ng/mL Frequencies	ima[C]min < 1000 ng/mL Frequencies	Chi Square	*p*-Value
Block 1	AAGGA	0.777	55.0:15.0	88.0:26.0	0.786	0.772	0.048	8.27 × 10^−1^
	TTTTT	0.190	12.0:58.0	23.0:91.0	0.171	0.202	0.259	6.11 × 10^−1^
Block 2	AGAACC	0.995	70.0:0.0	111.0:1.0	1.000	0.991	0.628	4.28 × 10^−1^
Block 3	AGGG	0.957	66.0:4.0	110.0:4.0	0.943	0.965	0.507	4.76 × 10^−1^
	TTTT	0.043	4.0:66.0	4.0:110.0	0.057	0.035	0.507	4.76 × 10^−1^
Block 4	ACA	0.880	62.9:7.1	98.9:15.1	0.899	0.868	0.403	5.25 × 10^−1^
	TTT	0.060	3.0:67.0	8.0:106.0	0.043	0.070	0.575	4.48 × 10^−1^
	ATA	0.028	2.0:68.0	3.0:111.0	0.029	0.027	0.01	9.19 × 10^−1^
	TCA	0.017	1.1:68.9	2.1:111.9	0.015	0.018	0.024	8.77 × 10^−1^
	TTA	0.010	1.0:69.0	1.0:113.0	0.014	0.008	0.117	7.33 × 10^−1^
Block 5	**CAGAAC**	0.962	63.0:7.0	114.0:0.0	0.900	1.000	11.851	6.00 × 10^−4^
	TTTAAC	0.011	2.0:68.0	0.0:114.0	0.029	0.000	3.293	6.96 × 10^−2^
	TTTTAC	0.011	2.0:68.0	0.0:114.0	0.029	0.000	3.293	6.96 × 10^−2^
	TTTTTT	0.011	2.0:68.0	0.0:114.0	0.029	0.000	3.293	6.96 × 10^−2^
Block 6	AAAA	0.886	62.0:8.0	101.0:13.0	0.886	0.886	0.000	9.96 × 10^−1^
	TTTA	0.076	5.0:65.0	9.0:105.0	0.071	0.079	0.035	8.52 × 10^−1^
	TTTT	0.022	2.0:68.0	2.0:112.0	0.029	0.018	0.248	6.19 × 10^−1^
Block 7	CAACAAA	0.989	69.0:1.0	113.0:1.0	0.986	0.991	0.123	7.26 × 10^−1^
	TTTTTTT	0.011	1.0:69.0	1.0:113.0	0.014	0.009	0.123	7.26 × 10^−1^

## Data Availability

CIC Hôpital Saint-Louis Paris France.

## Supplementary Materials

The following supporting information can be downloaded at: https://www.mdpi.com/article/10.3390/pharmaceutics16060834/s1, Figure S1: Principal component analysis (PCA); Figure S2: Manhattan plot for associations with plasma imatinib levels showing the log10(*p*-value) for individuals SNPS; Figure S3: Haplotypes of four genes with two or more SNPs in the Table 2. Analysis of SNPs located in NIPBL, BPGM, TCTN2 and EXOC6B genes associated with Ima[C]min in CML patients (Visualization of SNP genotypes with Haploview Software); Figure S4: Distribution of DEGs with non-corrected and BH-Adjusted *p*-values; Figure S5. Volcano plot with log2foldchange and -log10 p-values for the differentially expressed genes (DEGs) in patients with ima[C]min > 1000 ng/mL (*n* = 20) versus those with ima[C]min < 1000 ng/mL (*n* = 41). Figure S6: Gene expression profiling analysis. Tables S1.1 and S1.2: SNPs associated with ima[C]min greater than 1000 ng/mL; Tables S2.1 and S2.2: SNPs identified using linear regression analysis; Tables S3.1 and A3.2: SNPs associated with ima[C]min identified both by binary and by linear regression analysis; Table S4: SNPs identified by linear regression analysis with *p*-value ≤ 10^−4^ and mean depth of sequencing greater than 4 reads; Table S5: Excess of heterozygosity rates across a number of SNPs based on individual ima[C]min; Table S6: Differentially expressed genes (DEGs) associated to ima[C]min identify by RNA-seq; Table S7: Distribution of Adverse Events (AE) in function of ima[C]min.
