# Supplementary material for: Constitutional DNA Polymorphisms Associated with the Plasma Imatinib Concentration in Chronic Myeloid Leukemia Patients
Source: Pharmaceutics. 2024 Jun 19;16(6):834. doi: 10.3390/pharmaceutics16060834 (PMC11207966; doi:10.3390/pharmaceutics16060834)
Supplement: Supplementary file 1 [file pharmaceutics-16-00834-s001.zip › Supplemental Methods and Figures.pdf]

pharmaceutics

Article

Constitutional DNA Polymorphisms Associated with Plasmatic Imatinib Concentration in Chronic Myeloid Leukemia Patients

Supplemental Methods and Data.

Measurement of plasmatic imatinib concentration. Initial ima[C]min was evaluated after 12 weeks of treatment at 400 mg/daily at the time of arm assignation in the Optim-matinib study.
Ima[C]min was centrally determined by chromatography-tandem mass spectrometry as previously described [1]. Briefly, after a liquid–liquid extraction, imatinib and its deuterated internal standard were eluted on an XTerra RP18 column with a gradient of acetonitrile–ammonium formiate buffer 4 mmol/L, pH 3.2. Imatinib was detected by electrospray ionization mass spectrometry in multiple reaction-monitoring mode. The calibration curves were linear over the range 10–5000 ng/mL. The limit of quantification was set to 10 ng/mL.

Whole Exome Sequencing (WES)
- *DNA extraction.* Constitutional DNA from PBMCs samples was extracted from 114 patient included in OPTIM-imatinib trial following standard methods at the Hematology and Molecular Biology Department of Saint-Louis Hospital in Paris.
After validation by the CNRGH (Centre National de Recherche en Génomique Humaine) 100 DNA samples were sequenced using paired-end (2 × 150-bp) technology on an Illumina HiSeq 4000 platform at the CNRGH following the manufacturer's protocol. Quality control during sequencing (e.g. intensity, Q30, phasing/prephasing/base proportion) were considered.
- *Next-generation sequencing*. DNA samples from 100 CML patients treated by imatinib were enriched for exomic regions according to Agilent's SureSelect Human All Exon Kit protocol V6 (Agilent Technologies). The number of sequences produced per sample will enable mean coverage of nearly 100 to 120x (sequencing in pools of 6). All the samples passed the sequencing quality thresholds.
- *Read alignment*. It can be noted that bioinformatic analyses of WES as well as gene expression sequencing data were performed at Fondation Jean Dausset-CEPH (Centre d'Étude du Polymorphisme Humain, Paris, France). The data files were processed using bioinformatics tools. Sequencing reads (fastq files) were aligned to human reference genome GRCh38/hg38 (http://hgdownload.soe.ucsc.edu/goldenPath/hg38/bigZips/hg38.fa.gz) using BWA-mem (Burrows-Wheeler Aligner, version 0.7.17) to produce the sequence alignment file (bam files).
- *Post-alignment Processing*. The alignment was followed by assigning all reads in a file to a single new read-group using "AddOrReplaceReadGroups" command line from Picard tool (http://picard.sourceforge.net, picard version 2.25.0). The PCR (polymerase chain reaction) duplicates were marked before variant calling, based on their identical 5′ mapping coordinates and orientation on the genome with the purpose of eliminating PCR-introduced bias due to uneven amplification of DNA fragments (MarkDuplicates command from Picard tool program). The variant calling was carried out using HaplotypeCaller from Genome Analysis Toolkit (GATK, version 4.2.0.0) following the « Genomic Variant Call Format » (GVCF) workflow which is more suited for scalable variant calling i.e. allows incremental addition of samples for joint genotyping. The CombineGVCFs tool was applied to combine multiple single sample GVCF files, merging them into a single multi-sample GVCF file followed by the « GenotypeGVCFs tool » to generate vcf file. HaplotypeCaller program conducts a local de novo assembly of aligned reads prior to indel calling, which demonstrates to greatly improve the quality of indel calls. HaplotypeCaller simultaneously detects SNVs, indels, and some SVs with increased accuracy by performing a local de novo assembly of the aligned reads.

Association Studies
We have selected only bi-allelic variant (n=10 897 600) for Imatinib samples. A standard data quality control protocol was carried out before the association studies. The objective is to identify and remove biased DNA and marker samples. We used Plink v1.90b3f software, a SNP data processing tool, to perform evaluations of the Hardy Weinberg equilibrium failure rate, as well as the call rate. We thus excluded a total of 859 810 variants in Imatinib population because of the HWE test < 10^{-6} and 9 681 438 variants that have a call rate lower than 0.8. The total number of variants retained for the association study is equal to 1 170 455 for the Imatinib population of 100 patients.

In order to explore the population stratification of DNA variants, we performed a principal component analysis (PCA) by running Plink software using the HGDP-CEPH Diversity Panel which is a resource of 1,063 lymphoblastoid cell lines (LCLs) from 1,050 individuals in 52 world populations, banked at Jean Dausset-CEPH Foundation in Paris.
Single nucleotide polymorphisms (SNPs) shared by the two datasets (HGDP-CEPH and CML patients), after reducing the linkage disequilibrium (LD), were used to perform the PCA analysis. The principal components are based on a subset of seven HGDP population groups, and the CML individuals have been projected onto those PCAs. The top two principal components of the variance-standardized relationship matrix (PCA1 and PCA2) were extracted. The ancestry of the patients were characterized and only the main group of European individuals was included. Therefore, PCA allowed us to exclude 8 outliers from the association analyses. The remaining 92 samples (65 men and 27 women) that pass filters and QC (quality control) were included in association analysis (**Figure S1)**.

- Association analysis in binary mode. The Imatinib concentration was considered in binary form to perform association study using a Fisher's exact test. Two group of CML patients were compared in the association study: the first one consists of 35 patients with plasmatic imatinib concentration > 1000 ng/ml and the second of 57 patients with plasmatic imatinib concentration < 1000 ng/ml.

- Linear regression analysis. Quantitative trait such as plasmatic Imatinib concentration was tested for association with the genetic variants, using Plink software considering either asymptotic (likelihood ratio test and Wald test) or empirical significance values. The standard linear regression was performed by estimating the additive genetic model (additive effects of SNPs), i.e. dose-dependent effect of the minor alleles. The effect size of single SNP upon imatinib levels was estimated by univariate linear regression implemented into PLINK software, where the beta coefficient represents the estimated change in the dependent variable (SNP genotype) for a one-unit change in a predictor variable ima[C]min, while holding all other predictors constant. The beta coefficient represents the strength of the relationship between each predictor variable ima[C]min) and the dependent variable (SNP genotype) and the direction of the relationship (positive or negative).

- RNA seq Analysis

- Sample quality assessment. Initial quality control was performed at the National Institute of Health and Medical Research (INSERM, Paris, France). RNA isolates were quantified using spectrophotometry with NanoDrop 2000 (Thermo Scientific). The degree of sample contamination by nucleic acids, proteins and other contaminants was evaluated by determining the ratios of sample absorbance at 260 and 280 nm (A260/280). Values ≥1.8 were considered "pure" and acceptable for downstream molecular analyses.
In addition, complete RNA quality control on each sample was performed at CNRGH (Centre National de Recherche en Génomique Humaine, Institut de Biologie François Jacob, Evry, FRANCE). RNA concentrations were measured by UV quantification on a NanoDrop™ 8000 spectrophotometer (in duplicate). Indeed, RNA quality was tested using RNA Integrity Number (RIN) measurements by running a sample aliquot on a Bioanalyzer 2100 from Agilent, using the RNA6000 Nano Labchip kit (#5065-4476, Agilent Technologies, Inc., Santa Clara, CA). Selected samples for RNA sequencing were of good quality (no DNA contamination, no important degradation, RIN value > 7) and had concentration between 30-150 ng/µL.

- RNA library preparation and sequencing

RNA sequencing was performed at CNRGH. Libraries have been prepared using the "TruSeq stranded mRNA" Kit from Illumina, which selects polyA+ RNAs as a first step of library preparation and allows to get a clear view of the protein-coding transcriptome with strand-specific information. An input of 1 µg total RNA was used for all samples, and libraries were prepared on an automated platform, according to manufacturer's instructions. Library quality and concentration have been checked by LabGx (Perkin Elmer) and sample libraries have then been pooled before sequencing to reach the expected sequencing depth. Sequencing has been performed on an Illumina HiSeq4000 as paired-end 101 bp reads, using Illumina sequencing reagents. Libraries were pooled by 6 samples per lane, corresponding on average to 40 to 50 million sequenced fragments (or 80 to 100 million total reads).
The raw sequencing data was stored in FastQ format.
Quality control of RNAseq data was performed at CNRGH. Fastq files have been be processed by in-house CNRGH tools in order to assess quality of raw and genomic-aligned nucleotides. The following steps have been performed on a random selection of 2 x 10 million reads including the following steps : *(i)* a cleanup of FASTQ files using Trimmomatic

tool in order to determine the percentage of reads remaining after the removing of adapters (sequencing and multiplexing) and lower quality sequences; *(ii)* an alignment on the reference genome (Hisat2) as well as on the transcriptome and the ribosomal RNA (Bowtie2); *(iii)* the use of RSeqC and picardTools tools in order to generate quality metrics such as the percentage of "mapping" on the genome and transcriptome, the percentage of duplicate sequences, the percentage of ribosomal RNA (rRNA) and the total number of sequences.

- RNA-seq data processing for expression analysis

Cleaned reads (FASTQ files) obtained for each sample were aligned to the human reference genome GRCh38_r79 using STAR (version 2.5.3a) [2, 3]. (*"Genome Reference Consortium, human version 38"* (ftp://ftp.ensembl.org/pub/release-79/fasta/homo_sapiens/dna/Homo_sapiens.GRCh38.dna.chromosome.{1..22}.fa.gz/ ftp://ftp.ensembl.org/pub/release-79/fasta/homo_sapiens/dna/Homo_sapiens.GRCh38.dna.chromosome.{MT,X,Y}.fa.gz/ and gene annotation file GRCh38.79.gtf (ftp://ftp.ensembl.org/pub/release-79/gtf/homo_sapiens/Homo_sapiens.GRCh38.79.gtf.gz). We used for downstream analyzes the gene counts generated by STAR.

The differential expression analysis was carried out at gene and transcript levels, between 2 conditions (normal skin *versus* damaged skin (Morphea/Lichen) using *DESeq2* package (version: 1.26.0) [4] implemented in R (version R 3.4 available on the Bioconductor website: https://support.bioconductor.org) with its graphical user interface Rstudio (Version 2023.09.0, Build 463). *DESeq2* enables the identification of differentially expressed genes (DEGs) whose expression abundance is significantly increased (up-regulated) or decreased (down-regulated) in a pathological condition compared to a control condition. The results of the differential expression were defined for each gene in fold change (FC), p-value (estimated by Wald test) and adjusted p-value (padj) computed by Benjamini-Hochberg calculation to correct for multiple testing [5]. The obtained DEGs were identified according to the ENSEMBL nomenclature and matches in "hgnc_symbol" were also used.

- Pathway enrichment analyzes of DEGs

The functional analyzes of the DEGs making it possible to identify enriched biological pathways between the two patient groups (lichenous and morphea), were performed at CEPH using "Gene Set Enrichment Analysis" (GSEA).

The GSEA developed at the Broad institute (http://www.broad.mit.edu/gsea/index.jsp) allows the functional characterization of differentially expressed transcripts in one condition *versus* another condition using biological pathway annotation. Data from normalized read counts per gene obtained by *DESeq2* were used as input to GSEA. GSEA calculates an enrichment score (ES) using a weighted Kolmogorov–Smirnov-like statistic.

GSEA software and molecular signatures databases (MSigDB and blood transcriptional modules (BTMs)) were used for this analysis to determine prior-defined sets of genes that showed statistically significant, concordant differences between ima[C]min > 1000 ng/ml group and ima[C]min < 1000 ng/ml group. The GSEA analysis of normalized data showed the top 50 ranked overlap genes between CML patients with ima[C]min > 1000 ng/ml and those with ima[C]min < 1000 ng/ml revealed a dis-tinct on/off switch of genes, suggesting a pattern of upregulated/downregulated genes associated with ima[C]min > 1000 ng/ml lines (**Figure S6**). Our analysis detected 233 and 15 gene sets respectively when MSigDB and BTMs were used (data not shown) with NES > 1.8. The top ten significant and relevant gene sets identified and their GSEA statistics are provided in Supplementary Tables A8. Among the enriched gene sets, those relating to platelet activation, myeloid cells and monocytes, and bone marrow are the most important (Supplementary Tables A8).The statistical significance (nominal P value) of the ES was estimated by using an empirical phenotype-based permutation test procedure that preserves the complex correlation structure of the gene expression data. It first normalizes the ES for each gene set to account for the size of the set, yielding a normalized enrichment score (NES). NES reflects the degree of overrepresentation of a set of genes in a list of ranked genes. GSEA also controls the proportion of false positives by calculating the false discovery rate (FDR) corresponding to each NES; it is computed by comparing the tails of the observed and null distributions for the NES (^{1}000 permutations). The latest gene sets collection of the "Molecular Signatures Database" v7.0. (MsigDB) (http://www.broadinstitute.org/gsea/msigdb) was used for the analysis. GSEA also makes it possible to draw up a "heatmap" highlighting the 100 most differentially expressed genes on the basis of the counting data of reads standardized by *DESEq2*. The selection criteria used for "pathways" are as follows: nominal p-values and the lowest False Discovery Rate (FDR) (< 5%) and values of NES ≥ 1.8. The threshold of significance of NES (≥ 1.8) has been defined arbitrarily. An enrichment graph was generated for each enriched channel, making it possible to visualize the enrichment score, namely the genes the most involved in the enrichment of the channel in question.

Supplemntal Figures

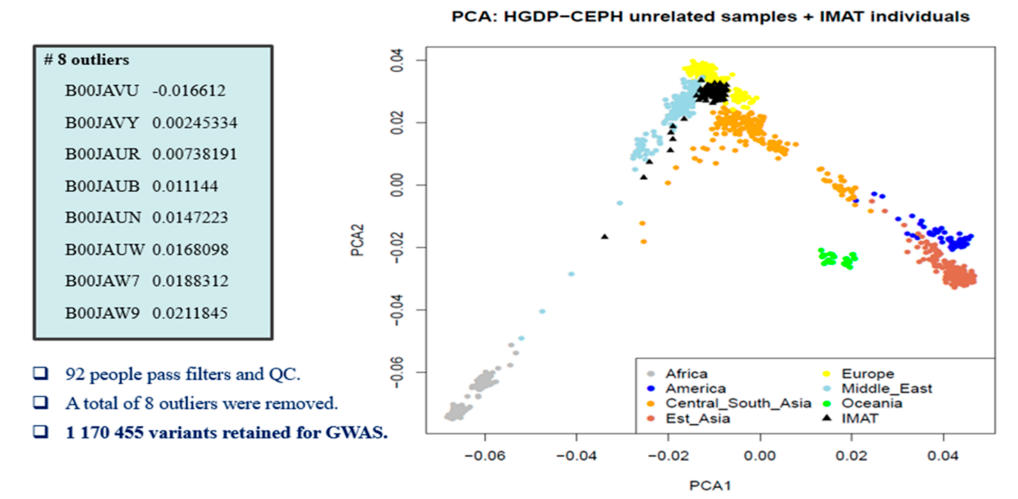

Figure S1. Principal Component Analysis (PCA). Plotted is PCA 1 (x-axis) vs PCA 2 (y-axis). Individuals are color-coded by continent of origin (Europe = yellow, Oceania = green, Africa = grey, America = blue, Est Asian = brown, Central South Asia= orange, 100 CML patients treated with Imatinib

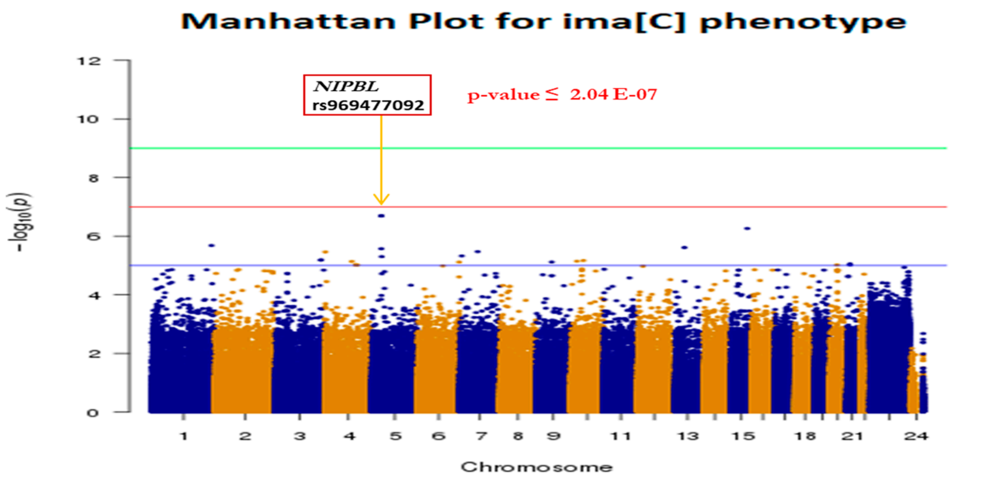

Figure S2. Manhattan plot for associations with plasma imatinib levels showing the log10 (p-value) for individual SNPs. The maximum suggestive association pointing to the *NIPBL* gene (p-value ≤ 2.04 E-07) located on chromosome 5 is indicated above the plot.

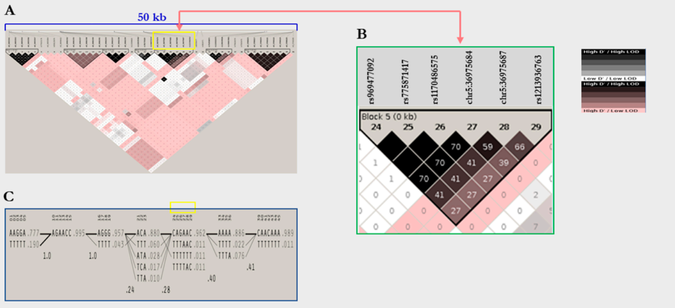

Figure S3.1. Haploview analysis for D′ pairwise measures of LD between *NIPBL* SNPs identified using linear regression in CML patients. (A) Linkage disequilibrium plot characterizing haplotype blocks using one SNP (rs969477092) located on *NIPBL* gene over a distance of 50 kb. SNPs that are in LD with rs969477092 are framed in yellow in block 5. **(B)** The numbers from 24 to 29 represent the selected SNPs and are framed in yellow. They are in LD. **(C)** Haplotypes display. The more frequent haplotype (CAGAAC) in block 5 associated with Ima[C]min> 1000ng/ml is framed in yellow.

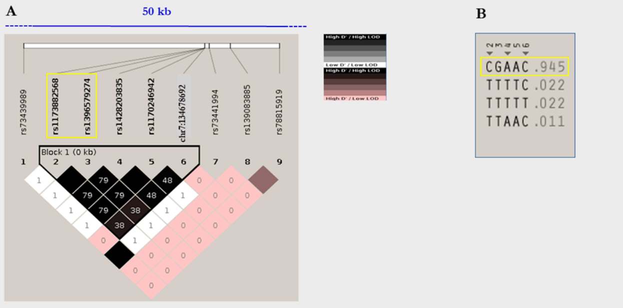

Figure S3.2. Haploview analysis for D′ pairwise measures of LD between *BPGM* SNPs identified using linear regression in CML patients. (A) Linkage disequilibrium plot characterizing haplotype blocks using one 2 SNPs (rs1173882568 and rs1396579274) located on *BPGM* gene over a distance of 50 kb. These 2 SNPs are framed in yellow. The numbers from 2 to 6 represent the SNPs that are in LD in the same block. **(B)** Haplotypes display. The more frequent haplotype (CGAAC) associated with Ima[C]min> 1000 ng/ml is framed in yellow.

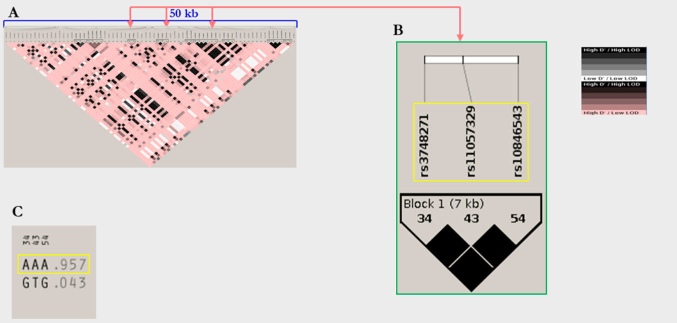

Figure S3.3. Haploview analysis for D′ pairwise measures of LD between *TCTN2* SNPs identified using linear regression in CML patients. (A) Linkage disequilibrium plot characterizing haplotype blocks using one 3 SNPs (rs3748271, rs11057329 and rs10846543) located on *TCTN2* gene over a distance of 50 kb. These 3 SNPs are located in blok 3, 4 and 6. **(B)** Numbers 34, 43 and 54 represent the selected SNPs which are framed in yellow and are in LD. (C) Haplotypes display. The more frequent haplotype (AAA) associated with Ima[C]min > 1000 ng/ml is framed in yellow.

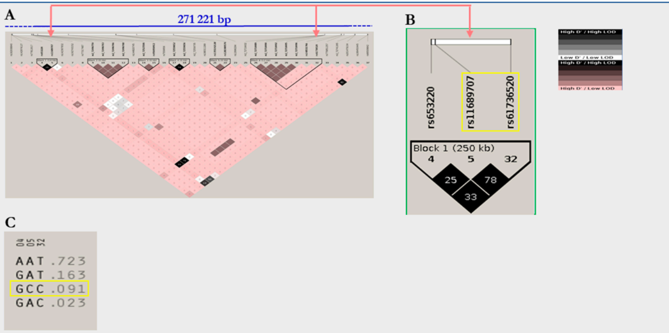

Figure S3.4. Haploview analysis for D′ pairwise measures of LD between *EXOC6B* SNPs identified using linear regression in CML patients. (A) Linkage disequilibrium plot characterizing haplotype blocks using 2 SNPs (rs61736520 and rs11689707) located on *EXOC6B* gene over a distance of 271 221 kb. These 2 SNPs that are located on block 1 and block 6 respectively are in LD. **(B)** The numbers 5 and 32 represent the selected SNPs that are framed in yellowy are in LD with rs653220. (C) Haplotypes display. The more frequent haplotype (GCC) associated with Ima[C]min> 1000 ng/ml is framed in yellow.

Figure S3. Haplotypes of four genes with two or more SNPs in the Table 2. Analysis of SNPs located in NIPBL, BPGM, TCTN2 and EXOC6B genes associated with Ima[C]min in CML patients (Visualization of SNP genotypes with Haploview Software). The same legend was applied to the 4 Haploview analyses (Figure S3.1–S3.4) i.e. the numbers within the squares represent the r2 (r-squared) scores for pairwise LD. D′ values and confidence levels (LOD) are represented as black for D′ = 1, LOD ⩾ 2; dark and light brown for D′ = 1, LOD < 2; white and rose for D′ < 1, LOD < 2.

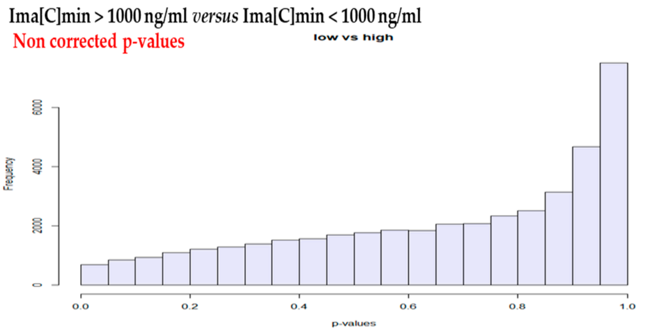

Figure S4.1. Distribution of non corrected p-values when no DEGs were identified that meet our criteria (BH-adjusted p-values ≤ 0,05 and |log2FC (fold-change)| > 1). The distribution of p-values increased when p-values go towards 1 which is not expected.

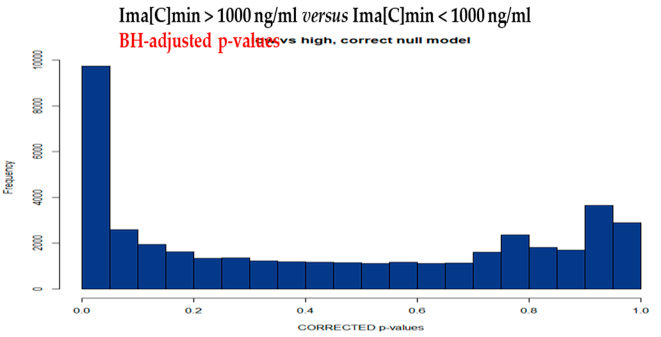

Figure S4.2. Distribution of new BH-adjusted p-values. Fdrtool returns the estimated null variance, as well as estimates of various other FDR related quantities and the p-values computed using the estimated null model parameters. New BH-adjusted p-values were calculated.

Figure S4. Distribution of DEGs with non-corrected (S4.1) and BH-Adjusted (S4.2) p-values

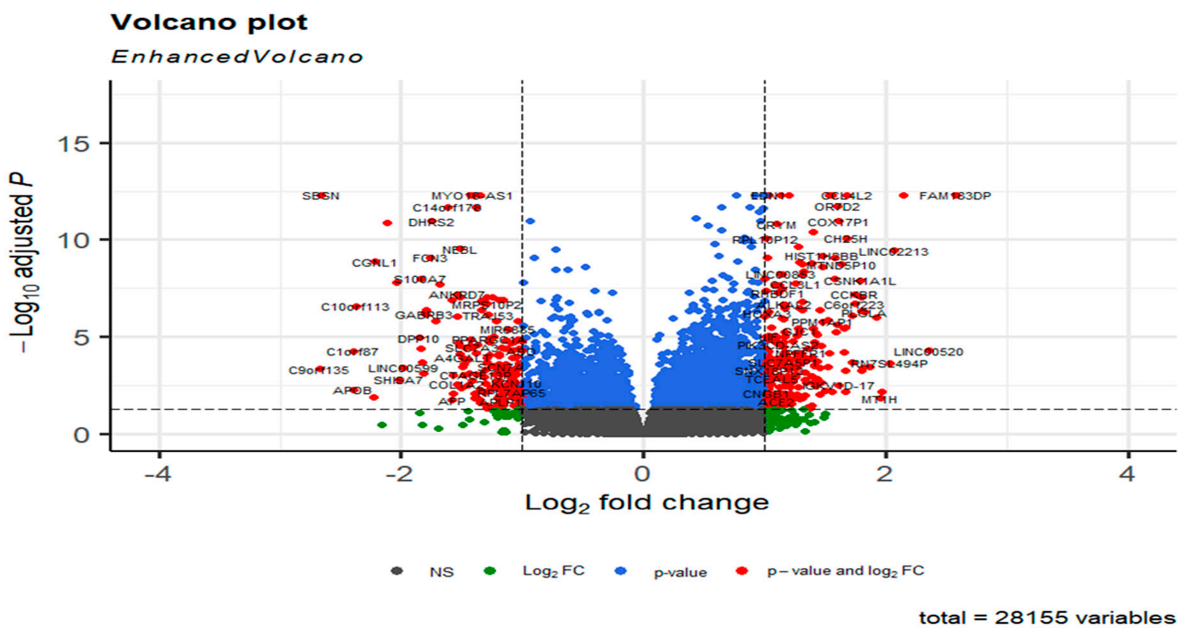

Figure S5. Volcano plot with log2foldchange and -log10 P values for the differentially expressed genes (DEGs) in patients with ima[C]min > 1000 ng/ml (n=20) versus those with ima[C]min < 1000 ng/ml (n=41). The most upregulated genes are towards the right, the most downregulated genes are towards the left, and the most statistically significant genes are towards the top.

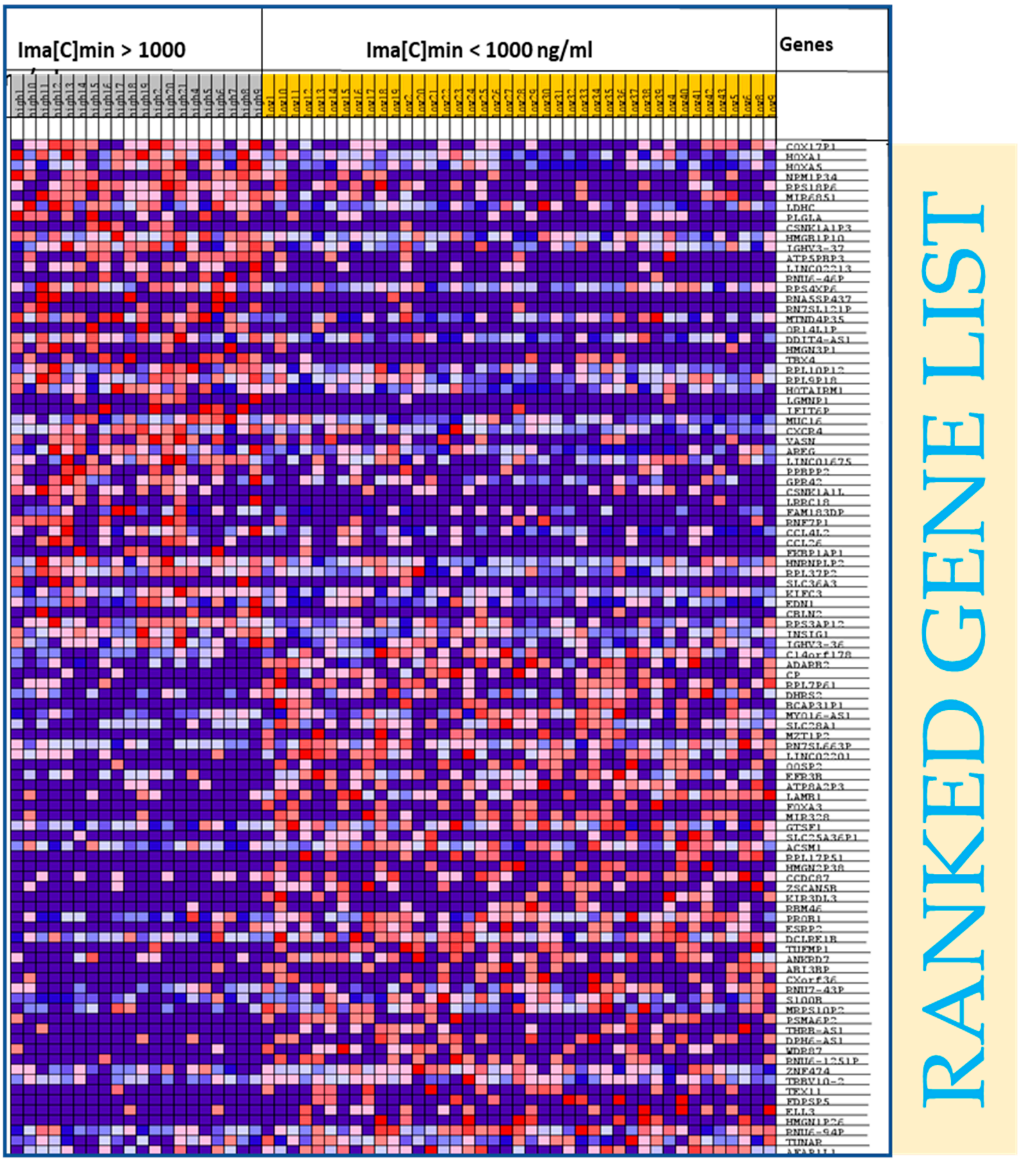

Figure S6. Gene expression profiling analysis. Heatmap of the top ranked genes generated using GSEA analysis on the common overlap genes between patients with Ima[C]min > 1000 ng/ml compared to patients with Ima[C]min < 1000 ng/ml. Red represents fold upregulation and blue represents fold downregulation.

References

1. Liu, Y.; Fang, B.; Jiang, J.; Wang, P. Clinical efficacy and safety of high-dose imatinib for chronic myeloid leukemia patients: An updated meta-analysis. *J. Can. Res. Ther.* **2016**, *12*, 23. https://doi.org/10.4103/0973-1482.191623.
2. Alghamdi, J.; Padmanabhan, S. Fundamentals of Complex Trait Genetics and Association Studies. In *Handbook of Pharmacogenomics and Stratified Medicine*; Elsevier: Amsterdam, The Netherlands, 2014; pp. 235–257, ISBN 978-0-12-386882-4.
3. Purcell, S.; Neale, B.; Todd-Brown, K.; Thomas, L.; Ferreira, M.A.R.; Bender, D.; Maller, J.; Sklar, P.; De Bakker, P.I.W.; Daly, M.J.; et al. PLINK: A Tool Set for Whole-Genome Association and Population-Based Linkage Analyses. *Am. J. Hum. Genet.* **2007**, *81*, 559–575. https://doi.org/10.1086/519795.
4. Park, J.-H.; Gail, M.H.; Weinberg, C.R.; Carroll, R.J.; Chung, C.C.; Wang, Z.; Chanock, S.J.; Fraumeni, J.F.; Chatterjee, N. Distribution of allele frequencies and effect sizes and their interrelationships for common genetic susceptibility variants. *Proc. Natl. Acad. Sci. USA* **2011**, *108*, 18026–18031. https://doi.org/10.1073/pnas.1114759108.
5. Barrett, J.C.; Fry, B.; Maller, J.; Daly, M.J. Haploview: Analysis and visualization of LD and haplotype maps. *Bioinformatics* **2005**, *21*, 263–265. https://doi.org/10.1093/bioinformatics/bth457.
